# Half-spaced substrate integrated spoof surface plasmon polaritons based transmission line

**DOI:** 10.1038/s41598-017-07799-0

**Published:** 2017-08-14

**Authors:** Jian Feng Zhu, Shao Wei Liao, Shu Fang Li, Quan Xue

**Affiliations:** 1 Electronic Engineering Department, State Key Laboratory of Millimeter Waves, City University of Hong Kong, Kowloon Tong, Hong Kong; 2 CityU Shenzhen Research Institute, Shenzhen, China; 3 Beijing Key Laboratory of Network System Architecture and Convergence, Beijing University of Posts and Telecommunications, Beijing, 100876 China

## Abstract

In this paper, a new spoof surface plasmon polaritons (SPPs) based transmission line (TL) with semi-open structure is proposed, which is implemented on a single-layer substrate with metallized via holes planted on a ground plane. The electromagnetic (EM) power propagates along it in the form of controlled slow surface wave, which is the same with its existing counterparts. The proposed TL can adjust the degree of EM energy confinement, and thus balance its performance in every characteristic, in particular attenuation and interference. As the TL is semi-open (i.e., EM energy distributes in the half space above the ground plane), it is less vulnerable to the nearby interference compared with its former counterparts, which are fully-open structure (i.e., EM energy distributes in the full space). Prototypes working at Ka band are fabricated and measured. Bianco-Parodi (BP) method is used to derive the attenuation of the proposed TL from the measured S-parameters. The proposed structure can be easily scaled for THz applications, which opens the door for future high performance THz components and systems.

## Introduction

Surface plasmon polaritons (SPPs) are the intensely localized surface waves that propagate along the dielectric-metal interface and decay exponentially in its transverse directions^1^. The most remarkable characteristic of SPPs is their electromagnetic fields can be well confined to the near vicinity of the interface. SPPs was believed to only exist in the optical regime for a long time until Pendry *et al*. demonstrate that they can also be observed in microwave frequencies on perforated conducting surfaces in 2004^2, 3^. Since then, plenty of work has been done to realize spoof SPPs in microwave, millimeter wave, and terahertz frequency bands^4–14^.

Transmission line (TL), as the foundation of all electronic components and systems, enables the interconnection of components and/or systems by delivering EM power/signal between them. More importantly, it is the very basic building block for distributed components at high frequency, such as filters, coupler and matching network, etc.^15^. Therefore, developing high performance TL is one of the fundamental challenges need to be addressed for THz technology^16, 17^. Various spoof SPP TLs with different geometries have been proposed^18–20^. Planar spoof SPP TLs, which are fully-open structure formed by a single periodically structured strip conductor etched on a substrate, were also designed^21, 22^. Because they can be easily fabricated by standard planar circuit technologies at low cost, these TLs are particularly attractive. The simplified form of planar spoof SPP TL with uniform strip conductor is also called planar Goubau line^23^. It was demonstrated that a planar spoof SPP TL can be used as an efficient channel for THz wave with low attenuation and low dispersion^24^. The propagation characteristics of EM wave propagating along a planar spoof SPP TL was investigated in THz band^25^. Several components, i.e. power divider and filter, have also been implemented on existing planar spoof SPP TLs in THz band^26, 27^.

Though low in attenuation and easy in fabrication in THz band, previous planar spoof SPP TLs have some intrinsic problems brought by their fully-open structures, which lack a common platform for circuit integration or fixture. Besides, as the structure is fully-open, the performance of previous planar spoof SPP TLs can be easily affected by the surrounding environment which is similar to ungrounded parallel stripline and coplanar waveguide. The strong mutual coupling makes these TLs inconvenient to be used in many complex circuit design and system integration, which are usually in a multilayered package form, especially when the waveguides at different layers that supporting multichannel spoof SPP are overlapping each other^28^. On the contrary, semi-open TLs with a ground plane, such as microstrip line, don’t have such problems. To overcome these disadvantages, substrate integrated spoof SPP TLs with semi-open structure might be a solution.

This paper proposed a new substrate integrated spoof SPP TL, whose structure is semi-open with unit cells planted on the ground plane. As an analogy, the proposed spoof SPP TLs are equivalent to microstrip line and ground coplanar waveguide, which have a common ground. While former spoof SPP TLs^21–23^ are equivalent to ungrounded parallel stripline and coplanar waveguide, which don’t have a common ground. As their characteristics can be easily affected by the components or structures in the vicinity, ungrounded coupled line and coplanar waveguide are much less used than microstrip line and ground coplanar waveguide. This conclusion may also apply to spoof SPP TLs, which justifies the advantages of the proposed spoof SPP TL. By tailoring the unit cell’s geometry, one can easily balance attenuation and EM field confinement for a specific application. Furthermore, the introduced ground plane makes the integration and implement of functional components much easier. This type of planar TL can be used to deliver THz signal and power efficiently, and more importantly, to conveniently build a series of novel THz functional components with high performance, such as antennas and filters, hence, lay the foundation of a new kind of compact high performance THz systems.

## Results

**Figure 1:**
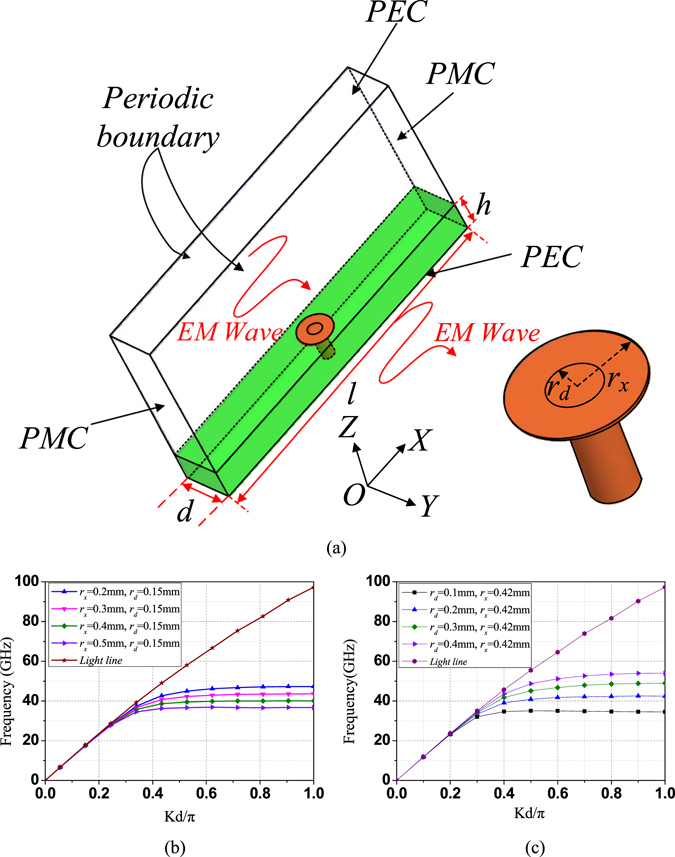
Schematic picture and dispersion diagrams of the proposed spoof SPP cell. (**a**) The proposed spoof SPP cell. *d* is the period of the unit cell, *h* is the height of the laminate and *l* is the width of the laminate. (*d* = 1.2 mm, *h* = 0.787 mm and *l* = 16 mm) (**b**) Dispersion diagram of the cells with different lengths of *r*_*x*_ when *r*_*d*_ is fixed at 0.15 mm. (**c**) Dispersion diagram of the cells with different lengths of *r*_*d*_ when *r*_*x*_ *is* fixed at 0.42 mm.

**Figure 2:**
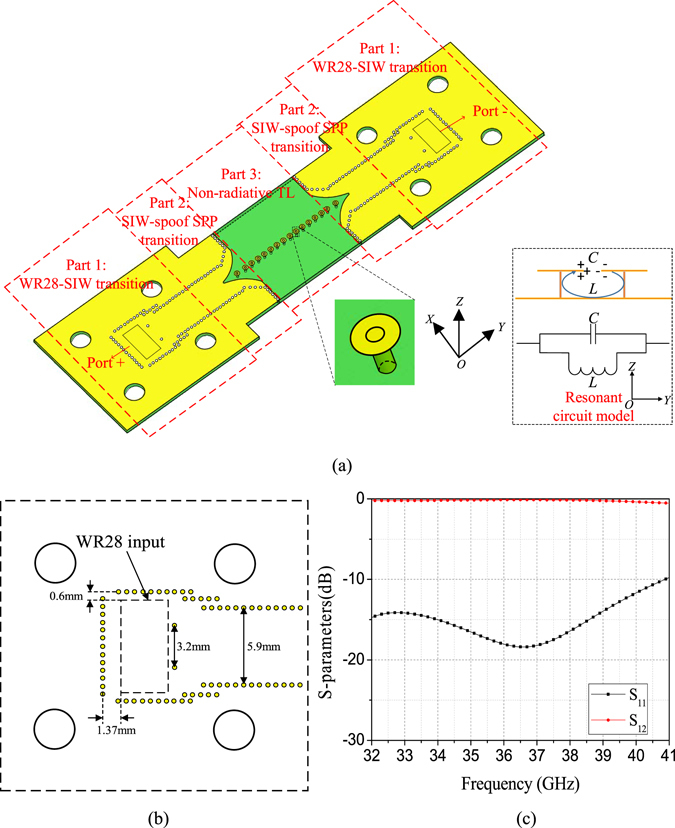
(**a**) The proposed substrate integrated spoof SPP TL that consists of three parts including Part 1: WR28-SIW transition, Part 2: SIW to spoof SPP transition, and Part 3: non-radiative TL. (**b**) Configurations of the WR28-to-SIW transition. (**c**) Simulated S-parameters of the WR28-to-SIW transition.

Figure 1(a) shows the proposed spoof SPP cell to derive the dispersion diagram that can be obtained by eigen mode analysis. The exterior region is bounded by periodic boundary in the longitudinal direction (OY-direction), and PMC boundary in the transverse direction (OX-direction), with PEC boundary above and below. The dispersion diagrams of the spoof SPP cell model are given in Fig. 1(b) and (c). It is seen that the dispersion diagrams of the cell gradually deviate from the light line when kd/π increases, and eventually asymptotically approach to their cutoff frequencies. Above the cutoff frequency, due to the physical intermodal between the forward and backward mode, the incident wave will be totally reflected^29^. The dispersion diagrams confirm that the electromagnetic wave is confined when it propagates along the proposed TL. Moreover, Fig. 1(b) and (c) also show the effects of the key parameters (i.e. the post diameter and the pad diameter) on the dispersion diagram. It is observed that the cutoff frequency increases with smaller pad diameter when the post diameter is fixed. While the cutoff frequency decreases with smaller post diameter when the pad diameter is fixed. The results of this analysis demonstrate that the cutoff frequency and the degree of EM energy confinement can be easily tuned by changing the geometrical parameters of the proposed structure. This simple array of closely spaced cells supporting spoof SPP can be understood by a version of high impedance surface (HIS) that possesses both capacitive and inductance components^30^. Its resonant mode can be explained with a simple circuit model as shown in Fig. 2(a), where the surface impedance is given by *Z*_*s*_ = *jωL/(1* − *ω* ^*2*^ *LC)*. This surface is inductive below the resonant (high impedance) frequency *ω*_*0*_ = *(LC)* ^*−1/2*^, and is thereby able to support a bound TM surface wave, which is cut off at the resonant frequency, analogous to the limiting surface plasma frequency of metals in the visible regime.

The configuration of the proposed substrate integrated spoof SPP-based TL with its waveguide excitation working at Ka band is given in Fig. 2(a). The proposed structure is printed on 0.787 mm thick Rogers 5880 laminate with dielectric constant of 2.2 and loss tangent of 0.0009. The thickness of the metal film is adopted as 0.018 mm. Substrate integrated waveguide (SIW)^31, 32^ is chosen as the excitation waveguide because the SIW can be easily integrated with the proposed TL in the same layer with a low profile. Therefore, the design in Fig. 2(a) consists of three parts: the standard WR28 waveguide-to-SIW transition, which can be regarded as a waveguide bend that is denoted as Part 1. Part 2 is the transition that is used to convert the SIW to spoof SPP mode. While Part 3 is the spoof SPP-based non-radiative TL with energy highly confined around it above the ground plane. For the part 1, the width of the SIW is chosen as 5.9 mm so that it works at the dominant mode. Two posts adjacent to the WR28 input are used to optimize the impedance matching between the WR28 waveguide and the SIW. The detailed geometry of the waveguide-to-SIW transition is shown in Fig. 2(b) while its simulated S-parameters are given in Fig. 2(c). It is observed that good matching is obtained and the insertion loss is optimized to better than 0.5 dB. Part 2 is the SIW to the spoof SPP transition. The top wall of the SIW is gradually expanded to excite the spoof SPP efficiently. The distances between the first three cells adjacent to the SIW are optimized to achieve good matching.

**Figure 3:**
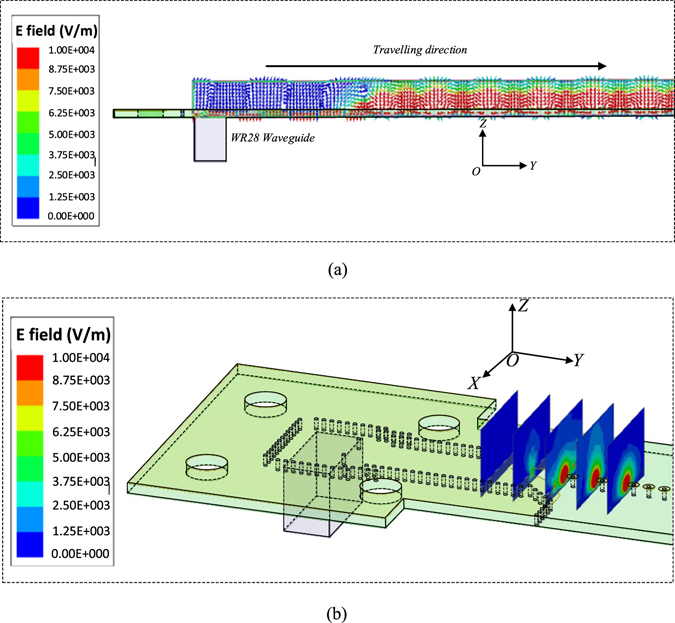
(**a**) Vector E-field distributions along the TL at YOZ-plane at 38 GHz. (**b**) |Ey| magnitudes of electrical field at planes perpendicular to the transition at 38 GHz.

The vector E-field distributions on the yoz-plane along the TL are given in Fig. 3(a). It is seen that the TE_10_ mode in the SIW has transformed to the TM polarized spoof SPP modes along the TL. Figure 3(b) shows the |Ey| magnitudes of the electrical field at planes perpendicular to the transition. It can be observed that the magnitudes of |Ey| of the field at SIW is zero and then gradual increase along the transition. Finally, TE_10_ in SIW is converted into TM mode supported by the spoof SPP structure.

**Figure 4:**
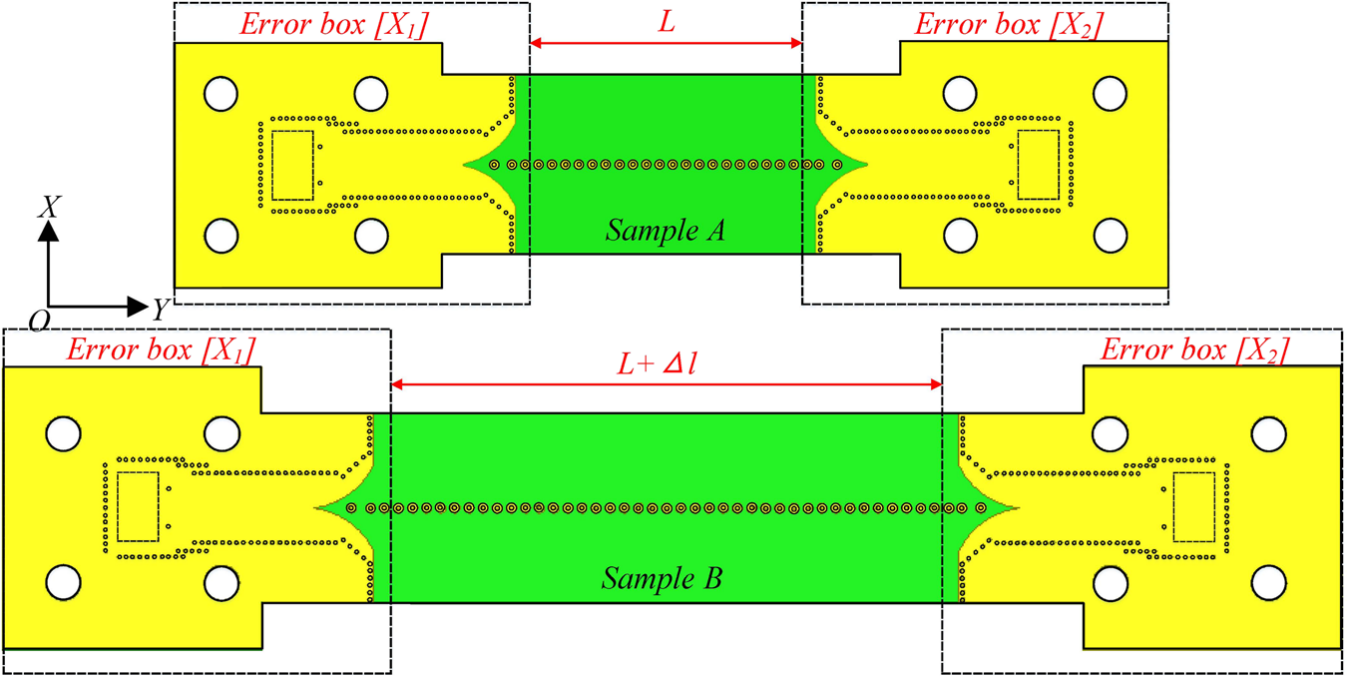
Bianco-Parodi measurement configuration for propagation constant determination.

The insertion loss of the proposed TL includes the conversion loss and the transmission loss. As only the transmission loss of the spoof SPP TL (part 3) is our interest, the impacts from the waveguide-to-SIW transition (part 1) and the SIW-to-spoof SPP transition (part 2) should be removed. Therefore, Bianco-Parodi (BP) method^33^ is used to de-embed the measured S-parameters and thus determine the propagation properties of the proposed TL. As shown in Fig. 4, two samples of the proposed spoof SPP TL, A and B, are designed with sample B 24 mm longer, or having 20 more spoof SPP cells than sample A. The waveguide-to-SIW transition and the SIW-to-spoof SPP transition are classified as the error box ([x_1_] and [x_2_]) and [x_1_] is the swap of [x_2_]. It can be shown that the propagate constant *γ* = *α* + *jβ* can be determined by the S-parameters of the two unequal length TL as follows^33^:

1 $$2\,\cosh (\gamma {\rm{\Delta }}L)=\frac{{S}_{21,a}^{2}+{S}_{21,b}^{2}+2{S}_{11,a}{S}_{11,b}-{S}_{11,a}^{2}-{S}_{11,b}^{2}}{{S}_{21,a}{S}_{21,b}}$$

Herein, *ΔL* equals to 24 mm (length of 20 cells). S_11, a_ and S_21, a_ are the S_11_ and S_21_ of sample A, respectively. S_11, b_ and S_21, b_ are the S_11_ and S_21_ of sample B, respectively.

**Figure 5:**
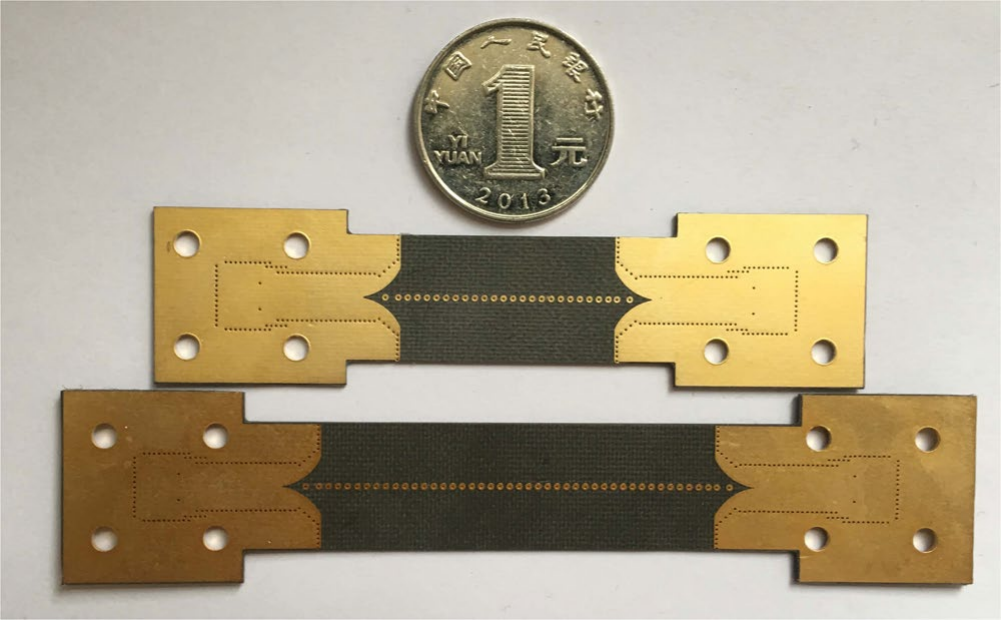
Fabricated prototypes of the two unequal length spoof SPP-based TLs (Sample A and B).

**Figure 6:**
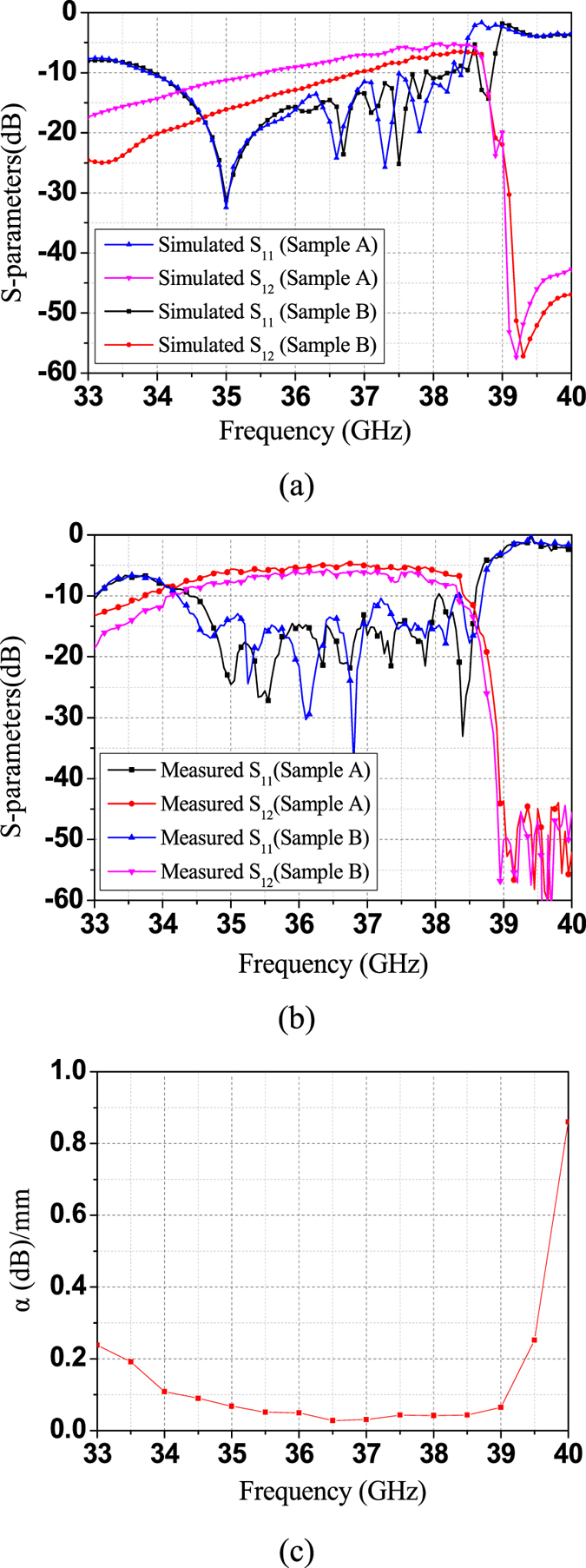
Simulated and measured S-parameters of the fabricated prototype and the calculated attenuation constant. (**a**) Simulated S-parameters of the sample A and B. (**b**) Measured S-parameters of the fabricated sample A and B. (**c**) Calculated attenuation constant based on the measured results.

**Figure 7:**
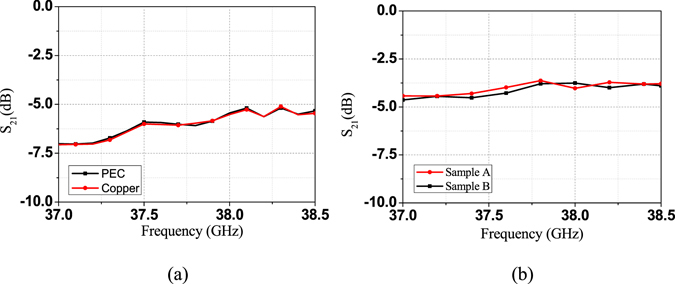
(**a**) Simulated S_21_ of the sample B using PEC and copper. (**b**) Simulated S_21_ for the sample A and sample B on a lossless dielectric substrate.

The fabricated prototypes of the two samples are shown in Fig. 5. Simulated and measured S-parameters of them are shown in Fig. 6(a) and (b), respectively. It can be seen that EM waves can travel along the spoof SPP TL below the cutoff frequency at about 39 GHz. Most of the insertion loss is from radiation loss of the SIW-to-spoof SPP transition. Thanks to the BP method, we can eliminate the impacts from the transition, and obtain attenuation constant of the proposed spoof SPP line as a function of frequencies, which is shown in Fig. 6(c). As can be seen, the calculated attenuation constant based on the measured results can be as low as 0.04 dB/mm, which includes the ohmic loss, the radiation loss and the dielectric loss. To evaluate the ohmic loss, we simulated the proposed TL with its copper metal replaced by PEC. The comparison of the S_21_ of using copper and PEC can be found in Fig. 7(a). The two transmission coefficient are almost identical, from which we can conclude that the ohmic loss is negligible. On the other hand, in order to evaluate the radiation loss, we simulated the proposed sample A and B on a lossless substrate, as shown in Fig. 7(b). It is seen that the insertion loss doesn’t vary much with the length of the TL. In other words, the radiation loss from the TL is small, which verifies that the energy is well-confined along the TL.

**Figure 8:**
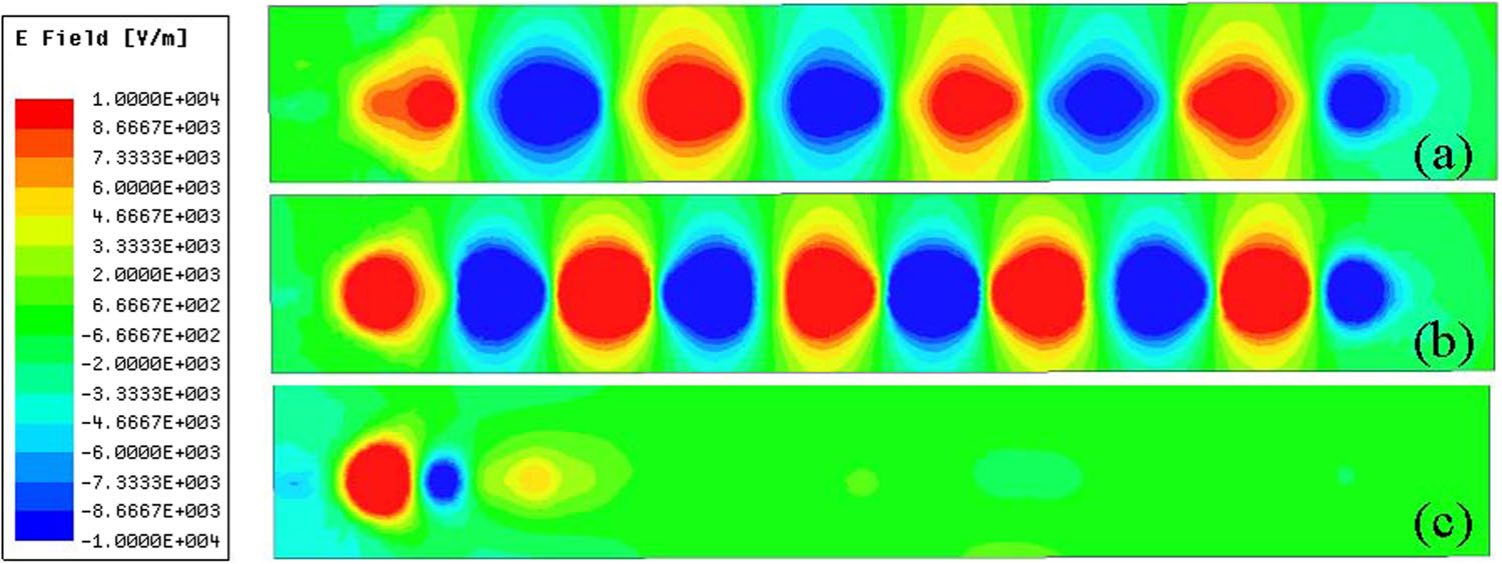
The simulated near-electric-field distributions on the xoy-plane which is 0.5 mm above the proposed TL. (**a**) Simulated results at 36 GHz. (**b**) Simulated results at 38 GHz. (**c**) Simulated results at 40 GHz.

To visually demonstrate the transmission feature of the proposed spoof SPP TL, the simulated near-electric-field distributions at different frequencies are shown in Fig. 8(a),(b) and (c), respectively. The observation plane is 0.5 mm above the proposed TL and the observation frequency points are chosen as 36, 38 and 40 GHz. From the near-field-distributions, one can clearly observe that the energy propagates along the TL steadily at the frequency of 36 and 38 GHz. While at 40 GHz, which is above the cutoff frequency, the EM wave cannot propagate.

**Figure 9:**
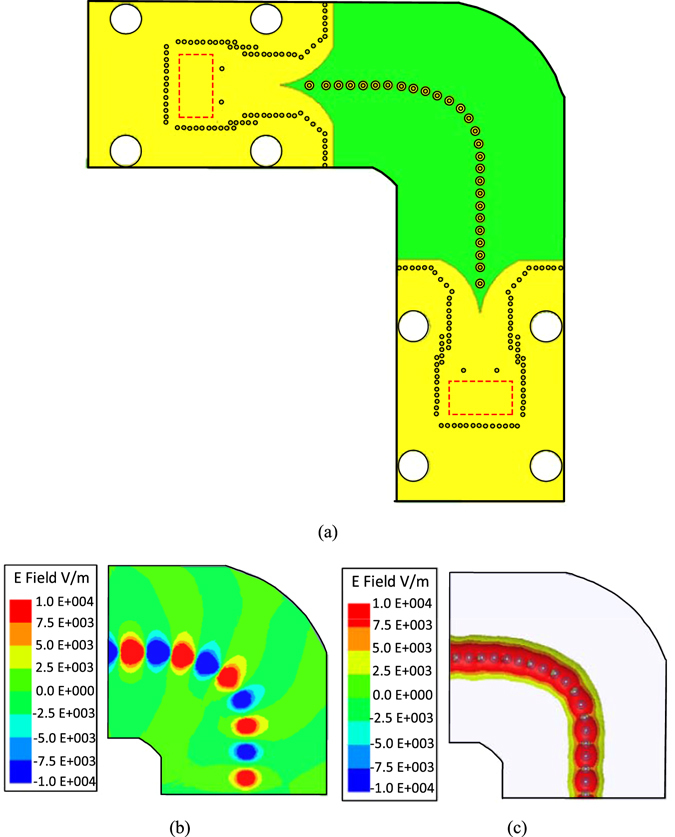
(**a**) Geometry of the 90° bend TL. (**b**) The simulated near-electric-field distributions at 38 GHz on the xoy-plane which is 0.5 mm above the proposed 90° bend TL. (**c**) Simulated electric-field distributions at 38 GHz in the substrate.

To further illustrate the transmission ability of the proposed spoof SPP transmission line, a 90° bend TL is designed, as shown in Fig. 9(a). The electrical field distributions along the bend TL are given in Fig. 9(b) and (c). Figure 9(b) presents the simulated near-electric-field distributions at 38 GHz with the observation plane 0.5 mm above the bend TL. Whereas Fig. 9(c) gives the E-field distributions in the substrate along the bend TL. It is observed that the energy is confined and propagate steadily along the 90° bend TL, which verifies the transmission capability of the proposed spoof SPP TL.

## Discussion

**Figure 10:**
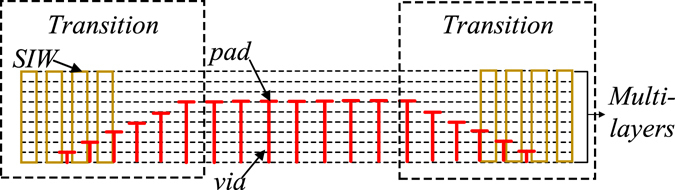
A possible configuration of the proposed TL with better transmission realized on multi-layered substrate technology.

Various fully opened spoof SPP TLs have been proposed in literatures^21–27, 34, 35^. The corrugated ultrathin plasmonic TLs for example, show very good energy confinement and plenty of functional devices based on it have been investigated in the past years. Nevertheless, they are still not very convenient to be used in complicated designs, such as multiplayer PCB based designs. Because there is no shield for the TL and there will be strong coupling between two TLs, especially when the waveguides at different layers that supporting multichannel spoof SPP are overlapping each other^28^. The proposed TL with ground, though shows relatively high transmission loss, can shield the energy at some certain layers and avoid the mutual coupling from each other. Besides, in some specific application, such as the point to point communication systems, directional antennas are usually required. Antennas with high directivity and high gain usually require a common ground and the antennas excited by the proposed TL might be a better option. It is also worth to mention that the conversion loss at the transition part is relatively high. Nevertheless, using gradually tapered cells and the SIW with higher height can improve the conversion loss. These approaches can be realized on several planar circuit technologies such as LTCC or multilayer PCB. A possible configuration of the transition with better transmission is shown in the Fig. 10.

In summary, a semi-open TL based on the substrate integrated spoof SPP is proposed and demonstrated numerically and experimentally. By adjusting the geometry of the spoof SPP cells, one can tune the energy confinement as well as the cutoff frequency. In addition, the structure can be easily scaled for the THz applications. Besides, as the TL is implemented using planar topology on a ground, it can be easily integrated with various mm-Wave and THz circuits and systems. More importantly, compared with its counterparts that are fully open, the proposed semi-open TL are more suitable for the THz circuits and system as the interference between layers can be reduced to the minimum, which opens a door for the future high performance THz components and systems.

## Methods

Numerical simulations are performed by ANSYS High Frequency Structure Simulator (HFSS) ver.16. The experimental structure is fabricated by using Rogers 5880 laminate with a dielectric constant of 2.2 and loss tangent of 0.0009. The thickness of copper is 0.018 mm with a conductivity of 5.8 × 10^7^ S/m. Agilent vector E8361A is used to measure the S-parameters.
